# Morphometric studies on the appendicular bony skeleton of the ostriches (*Struthio Camelus*)

**DOI:** 10.1186/s12917-023-03665-6

**Published:** 2023-08-04

**Authors:** Menna Allah M. Kassem, Reem R. Tahon, Karim M. Khalil, Medhat A. El-Ayat

**Affiliations:** Anatomy and Embryology Department, Faculty of Veterinary Medicine, Cairo University, Giza, Egypt

**Keywords:** Couple patellae, Femur, Morphometric, Ostrich, Pedal digits, Scapulo-coracoid, Tarsometatarsus, Wing

## Abstract

**Background:**

Morphometric study of the bony elements of the appendicular skeleton in the ostrich was fully described and identified. The appendicular skeleton included the bones of the pectoral girdle, the wing, the pelvic girdle and the pelvic limb.

**Results:**

The shoulder girdle of the ostrich included the scapula and coracoid bones. The scapula appeared as a flattened spoon-like structure. The coracoid bone appeared quadrilateral in outline. The mean length of the scapula and coracoid (sternal wing) were 15.00 ± 0.23 and 10.00 ± 0.17 cm, respectively. The wing included the humerus, ulna, radius, radial carpal bone, ulnar carpal bone, carpometacarpus and phalanges of three digits. The mean length of the humerus, radius, and ulna were 33.00 ± 0.46, 10.50 ± 0.40 and 11.50 ± 0.29 cm respectively. The carpometacarpus was formed by the fusion of the distal row of carpal bones and three metacarpal bones. Digits of the wing were three in number; the alular, major and minor digits. Os coxae comprised the ilium, ischium and pubis. Their mean lengths were 36.00 ± 0.82 cm, 32.00 ± 0.20 and 55.00 ± 0.2.9 cm, respectively. The femur was a stout short bone, that appeared shorter than the tibiotarsus. The mean length of the femur, tibiotarsus, and tarsometatarsus were 30.00 ± 0.23, 52.00 ± 0.50 and 46.00 ± 0.28 cm. Tibiotarsus was the longest bone in the pelvic limb. The fibula was a long bone (44.00 ± 0.41 cm) lying along the lateral surface of the tibiotarsus. The tarsometatarsus was a strong long bone formed by the fusion of the metatarsal (II, III, IV) and the distal row of tarsal bones. It was worth mentioning that metatarsal II was externally absent in adults.

**Conclusions:**

In the appendicular skeleton of ostrich, there were special characteristic features that were detected in our study; the clavicle was absent, the coracoid bone was composed of a sternal wing and scapular wing, the ulna was slightly longer in length than the radius. The coupled patellae i.e., the proximal and distal patella were observed; and the ostrich pedal digits were only two; viz., the third (III) and fourth (IV) digits.

## Background

Ostrich (*Struthio camelus*) is the largest and fastest living biped animal [1]. Ostrich is a ratite (flightless) bird from the order *Struthioniformes*, Suborder *Struthiones*, family *Struthionidae* and genus Struthio [2]. There were four subspecies of ostrich: the North African ostrich (*Struthio camelus camelus*), the Somali ostrich (*S. c. molybdophanes*), the Massai ostrich (*S. c. massaicus*), and the South African ostrich (*S. c. australis*) [2].

Compared to mammals, birds can stand, walk and run on two legs (bipedal), making the pelvic limb of birds more important [3]. The hind limbs had unique anatomical skeletal features [1]. The longest and strongest bone in mammals is the femur whereas in birds it is tibiotarsus [3]. The length of the tibiotarsus also indicates a bird's behavior, the walking birds have a longer tibiotarsus when compared to birds with running and swimming abilities [3]. The available literature describing the anatomy of the appendicular skeleton in ostriches seems to be meager and scanty. Hence the present study was planned to investigate the gross morphology and morphometry of different bony elements of the appendicular skeleton in ostrich.

## Materials and methods

Twelve male ostriches aged between 2–3 years and with an average weight of 120.00 kg, were obtained from Abu Rifai Ostrich Farm in the European countryside at Cairo-Alexandria Desert Road. Some of the obtained ostriches were collected after slaughtering for meat consumption then we used the bones for our study and other ostriches were died accidentally on the farm and we dissected them for obtaining the skeleton.

### For specimen preparation

Manual removal of skin, muscles and viscera with ordinary dissecting tools was performed.

### For cleaning the bones

Complete maceration by potassium hydroxide (KOH) method was used. The specimens were boiled for 2–3 h in 5% KOH and then washed with water to remove soft tissue and chemical residues [4].

### For the degreasing and bleaching process

For the degreasing process, the bones were soaked in acetone (100% concentration) for 2–3 days to remove the grease from the bones. The bones were washed with water to remove acetone residues. The bones were dried at room temperature before inserting them in bleaching solution. The bones were then soaked in 5% hydrogen peroxide for 3 days [5]. The bones were removed from the bleaching solution when they reached the desired white color, then they were soaked in water for two days to remove any chemical residues from the bones. The bones were dried in the air for 5 days.

The cleaned bones were photographed using a cannon digital camera, 16.1 MP, 4x. The nomenclature used in this study was that given by the *Nomina Anatomica Avium* [6].

The metric study was conducted on 12 cases using the digital Vernier caliper with values in millimeters (mm) and a weighing scale having calibration in milligrams for weighing each bone individually.

## Results

**Fig. 1:**
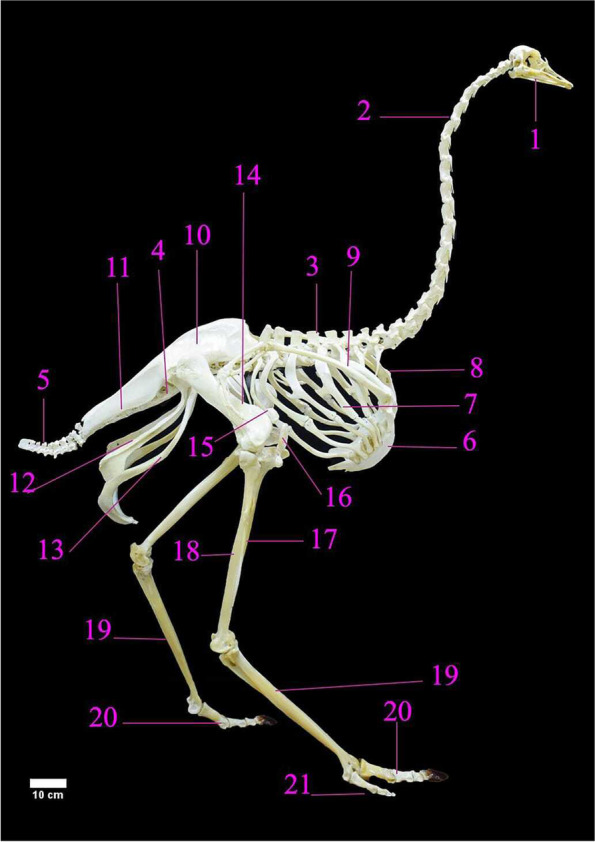
A Photograph showing the skeletal architecture of male ostrich (lateral view) of age 2 years and 3 months. 1. Skull. 2. Cervical vertebrae. 3. Thoracic vertebrae. 4. Synsacrum. 5. Caudal vertebrae. 6. Sternum. 7. Ribs. 8. Pectoral girdle. 9. Wing. 10. Preacetabular ilium. 11. Post acetabular ilium. 12. ischium. 13. Pubis. 14. Femur. 15. Proximal patella. 16. Distal patella. 17. Tibiotarsus. 18. Fibula. 19. Tarsometatarsus. 20. III digit. 21. IV digit

The ostrich skeleton was comprised of the axial and appendicular skeleton (Fig. 1). The axial skeleton was composed of the cranium, sclerotic ring, mandible, hyoid bone, vertebral column, ribs, and sternum. The appendicular skeleton contained the scapulo-coracoid, wing bones, os coxae and pelvic limb bones (Fig. 1).

**Fig. 2:**
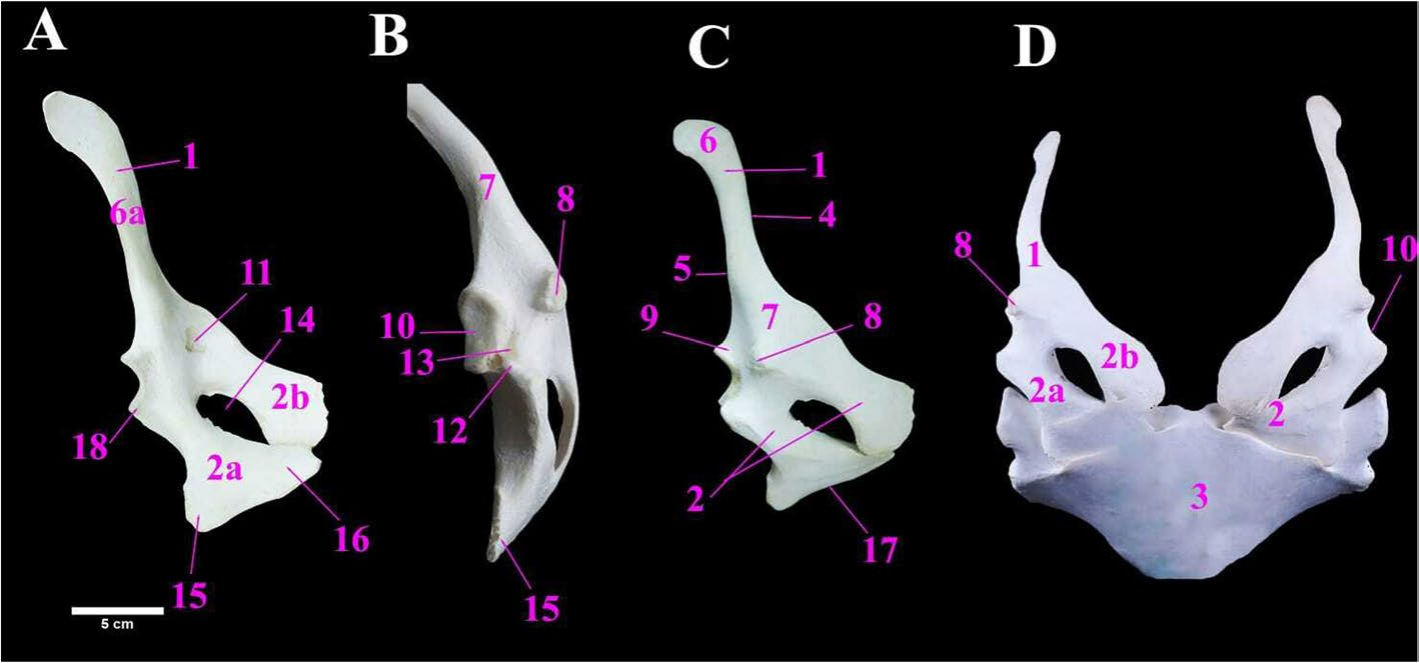
**A** Photograph showing the medial surface of the left Scapulocoracoid bone of male Ostrich, **B** The caudal view of right Scapulo-coracoid bone, **C** The lateral surface of right Scapulo-coracoid bone, and **D** The cranial view of both right and left pectoral girdles articulated with the sternum. 1.Scapula. 2.Coracoid. 2a. Sternal wing of coracoid. 2b. Scapular wing of coracoid. 3. Sternum. 4. Cranial border of scapula. 5. Caudal border of scapula. 6. The caudal end of scapula 6a. Blade of scapula. 7. Proximal extremity of scapula. 8. Acromion process. 9. Glenoid process of scapula. 10. Glenoid cavity. 11. Pneumatic foramen. 12. Acrocoracoid process. 13. Supracoracoid sulcus. 14.Coracoid fenestra. 15. Lateral angular process. 16. Medial angular process. 17. Sternal articular facet. 18. Glenoid process of coracoid

The pectoral girdle (shoulder girdle) of Ostrich was comprised of the scapulo-coracoid bones (Fig. 2A & C) while the clavicle was absent. The mean weight of the pectoral girdle was 65 ± 1.73 gm (Table 1). The scapulo-coracoid comprised a fused scapula and coracoid bone (Fig. 2D). We observed a supracoracoid sulcus (Fig. 2B). The scapula appeared as a flattened spoon-like structure (Fig. 2C), supported with an elongated narrow blade (Fig. 2A). The scapula was directed caudo-dorsally, slightly twisted medially (Fig. 2C). The scapula possessed two surfaces i.e., the lateral and medial surfaces (Fig. 2A), and two borders viz., the cranial and caudal borders (Fig. 2C). The lateral surface had proximally large semicircular acromion process and at its caudal end was slightly concave, ending at the level of the third rib. Medially, the blade of the scapula was slightly convex, and its proximal extremity had a large pneumatic foramen (Fig. 2A). The coracoid bone appeared quadrilateral in outline with two wings; sternal wingand scapular wing, separated by a coracoid fenestra (Fig. 2A). It had an acrocoracoid process (Fig. 2B) dorsal to the glenoid process of coracoid (Fig. 2A). The sternal wing of coracoid bone had an apex and the base, the apex articulated with scapula forming the glenoid cavity laterally, while the base had lateral and medial angular processes (Fig. 2A) which articulated with the sternum by the sternal articular facet (Fig. 2C). The supracoracoideus sulcus (Fig. 2B) was present between the acrocoracoid process and the glenoid process of the scapula. The proximal extremity of the scapula joined the sternal end of the coracoid bone to form the glenoid cavity (Fig. 2B & C) which was directed dorsolateral and articulated with the proximal extremity of the humerus.

**Table 1:** Metric data of scapulo-coracoid bone of ostrich (*Struthio camelus*)

Item of Description	Mean length (cm)	Mean width (cm)
**Scapula**	15.00 ± 0.23	4.00 ± 0.14
**Coracoid (scapular wing)**	10.00 ± 0.20	3.80 ± 0.11
**Coracoid (sternal wing)**	10.00 ± 0.17	5.00 ± 0.16

**Fig. 3:**
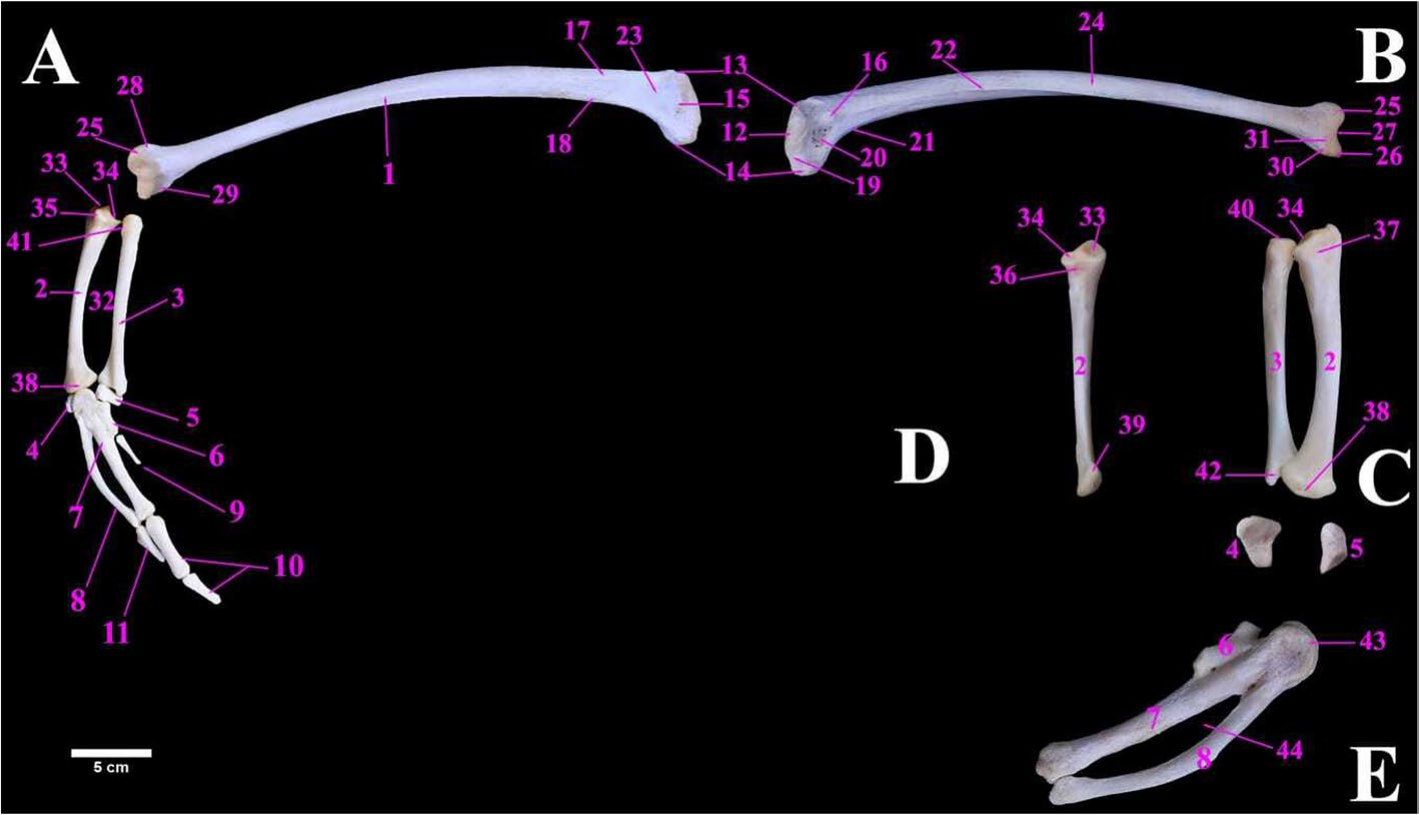
**A** lateral view of the male ostrich right wing bones, **B** medial view of right humerus, **C** medial view of right radius and ulna with separated radial and ulnar carpal bones, **D** cranial view of right ulna, **E** medial view (palmar) of carpometacarpus bone. 1. Humerus. 2. Ulna. 3. Radius. 4. Ulnar carpal bone (ulnare). 5. Radial carpal bone. 6. First metacarpal bone. 7. Second metacarpal bone. 8. Third metacarpal bone. 9. First (alular) digit. 10. Second (major) digit. 11. Third (minor) digit. 12. Head of humerus. 13. Dorsal tubercle. 14. Ventral tubercle. 15. Lateral tubercle. 16. Medial tuberosity. 17. Deltoid crest. 18. Bicipital crest. 19. Transverse sulcus. 20. Pneumotriciptal fossa with pneumatic foramen. 21. Intermuscular line. 22. A ridge. 23. Coracobrachialis impression. 24. Shaft of humerus. 25. Lateral condyle. 26. Medial condyle. 27. Intercondylar notch. 28. Lateral epicondyle. 29. Medial epicondyle. 30. Flexor process. 31. Olecranon fossa. 32. Interosseous space. 33. Dorsal cotyla of ulna. 34. Ventral cotyla of ulna. 35. Olecranon process. 36. Concave radial articular facet. 37. Brachialis impression. 38. Carpal trochlea of ulna. 39. Carpal tubercle. 40. Humeral cotyla of radius. 41. Ulnar articular facet. 42. Carpal articular facet of radius. 43. Carpal trochlea of carpometacarpus bone. 44. Interosseous space of carpometacarpus bone

The Ostrich wing was comprised of the humerus, ulna, radius, radial carpal bone, ulnar carpal bone, carpometacarpus and phalanges of three digits. The humerus (Fig. 3A) was the longest bone in the wings. Its mean weight was 116.00 ± 0.90 gm (Table 2). In resting condition, it was oriented parallelly to the first six ribs. It possessed a large proximal extremity, a curved shaft and a smaller distal extremity. The proximal extremity was composed of the head of the humerus, lateral tuberosity, medial tuberosity, and dorsal and ventral tubercles (Fig. 3A & B). The head of the humerus was semicircular in outline with a prominent neck for articulation with the glenoid cavity of scapulo-coracoid bone. The lateral tuberosity had a coracobrachialis impression. The dorsal tubercle was traced at the dorsolateral aspect of the scapula which continued distally as a deltoid crest (Fig. 3A), while the ventral tubercle continued distally as the bicipital crest (Fig. 3A). A transverse sulcus (Fig. 3A) was observed between the head and the ventral tubercle. Caudoventral to the head of the humerus, a pneumotricipital fossa (Fig. 3B) was present which was perforated by several pneumatic foramina. The distal extremity of the humerus had lateral and medial condyles (nearly equal in size and separated by an intercondylar notch), two epicondyles (lateral and medial) and a small olecranon fossa (on the medial aspect of the distal end). The long, cylindrical shaft of the humerus (Fig. 3B) was curved and possessed four surfaces namely the dorsal, ventral, lateral and medial surfaces. It had a medially placed intermuscular line and a large longitudinal ridge (Fig. 3B).

**Table 2:** Metric data of humerus bone of ostrich (*Struthio camelus*) P. proximal, D. distal, MS. Midshaft

Item of Description	Mean length (cm)	Mean width (cm)	Mean circumference of the shaft (cm)
**Humerus**	33.00 ± 0.46	2.60 ± 0.35 (P)	7.00 ± 0.25 (P)
		1.60 ± 0.32 (D)	6.00 ± 0.18 (MS)
		1.80 ± 0.30 (M)	5.00 ± 0.137 (D)

The Radius and ulna were separated by a wide interosseous space (Fig. 3A). They articulated with distal condyles of the humerus. The metric data of the forearm and manus bones of ostrich were shown in Table 3. The ulna (Fig. 3A & C) was slightly longer than the radius and had a cylindrical slightly curved shaft. The proximal extremities of both the radius and ulna possessed a dorsal and a ventral cotyla (Fig. 3D) for articulation with lateral humeral condyles (Fig. 3D), The ulna had a small olecranon process (Fig. 3A) laterally and a shallow brachialis impression medially (Fig. 3C). The distal extremity comprised a single carpal trochlea (Fig. 3D) of the ulna for ulnare articulation. It also had a pointed carpal tubercle cranially. The radius (Fig. 3A & C ) had a rod-like shaft that was thinner than the ulna. The proximal extremity possessed a humeral cotyla (Fig. 3C) and a concave ulnar articular facet (Fig. 3A) for articulation with the ulna. The distal extremity also had the carpal articular facet (Fig. 3C) for radiale articulation.

**Table 3:** Metric data of forearm and manus bones of ostrich (*Struthio camelus*)

Item of Description	Mean weight (gm)	Mean length (cm)	Mean width (cm)
**Radius**	8.00 ± 0.30	10.50 ± 0.40	1.50 ± 0.14
**Ulna**	14.00 ± 0.29	11.50 ± 0.29	2.00 ± 0.38
**Carpometacarpus**	8.00 ± 0.25	8.00 ± 0.30	2.50 ± 0.25
**Ulnar carpal bone**	1.00 ± 0.18	1.50 ± 0.34	1.00 ± 0.20
**Radial carpal bone**	0.50 ± 0.30	1.50 ± 0.20	0.70 ± 0.30
**First (Alular) digit**: one phalanx	0.50 ± 0.21	2.00 ± 0.38	0.30 ± 0.29
**Second (major) digit**: 1st phalanx	0.60 ± 0.15	3.50 ± 0.27	1.00 ± 0.32
**Second (major) digit**: 2nd phalanx	0.50 ± 0.20	2.30 ± 0.29	0.80 ± 0.19
**Third (minor) digit**: one phalanx	0.40 ± 0.25	2.30 ± 0.25	0.70 ± 0.13

The Carpal bones included two carpal bones: the radial carpal and ulnar carpal (Fig. 3A & C). The radial carpal bone was larger than the ulnar carpal bone. It was quadrilateral in outline while the ulnar had round borders. They articulated proximally with the radius and ulna, and distally with the carpometacarpus.

The Carpometacarpus bone was formed by the fusion of the distal row of carpal bones and the three metacarpal bones; the 1^st^ Alular, 2^nd^ (Major) and 3^rd^ metacarpal bone (Fig. 3 A & E). The alular metacarpus was short and fused with the second metacarpus, the second metacarpus was straight and the thickest, and the third metacarpus was strongly curved and fused to the proximal and distal ends of the second metacarpus leaving an interosseous space between of carpometacarpus bone (Fig. 3E). The carpometacarpus proximally had a carpal trochlea (Fig. 3E) for articulation with carpal bones.

Wing digits, three digits were observed in ostrich which articulated with the carpometacarpal bone. The first (alular) digit had one phalanx, the second (major) digit had two phalanges, and the third (minor) digit had one phalanx (Fig. 3A). The 1^st^ phalanx of the major digit was larger and thicker while the 2^nd^ phalanx was pointed and triangular.

**Fig. 4:**
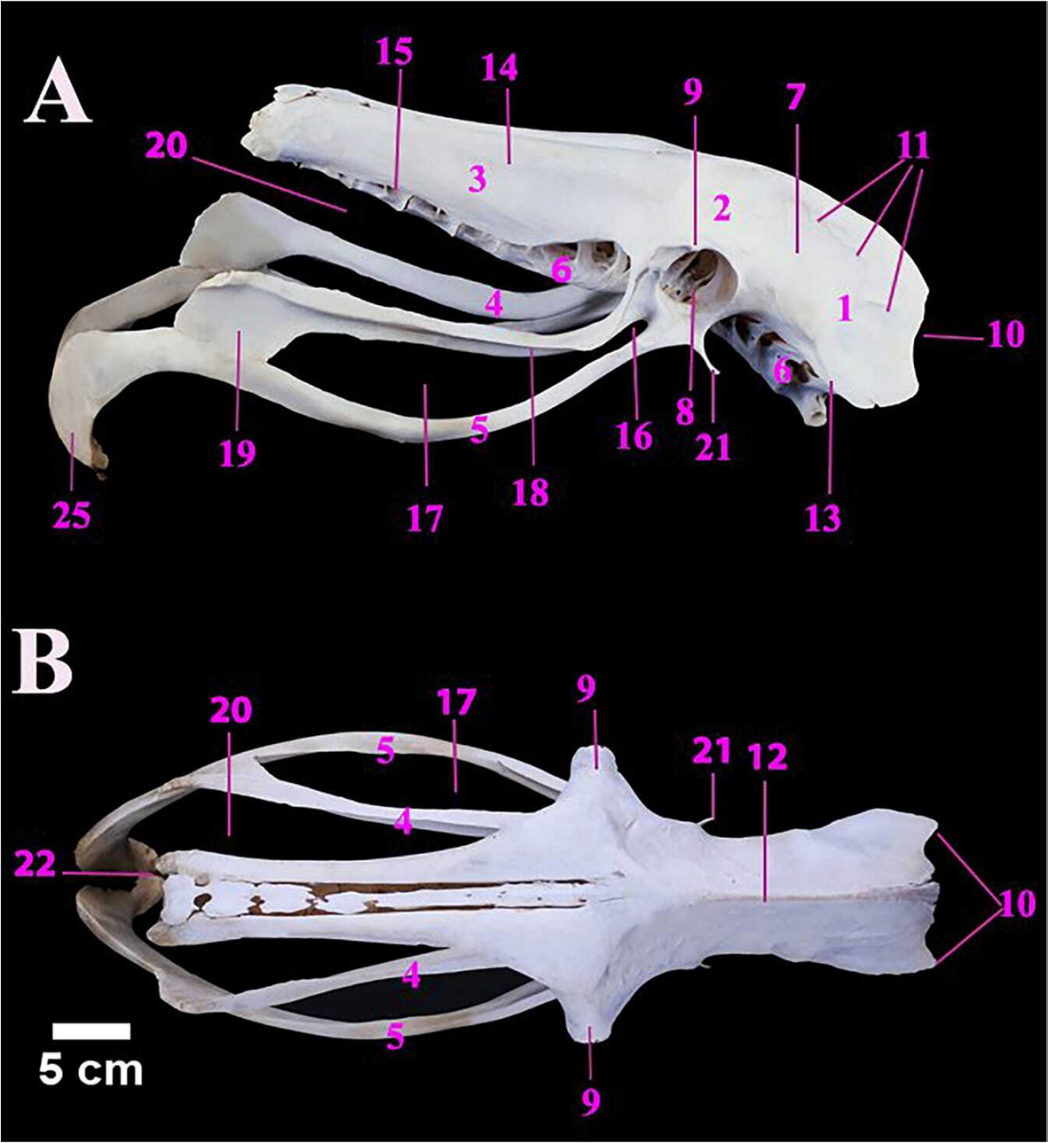
**A** & **B** photographs showing the lateral and dorsal view of the pelvic girdle of male Ostrich. Legend: 1. Preacetabular part of ilium. 2. Acetabular part of ilium. 3. Post acetabular part pf ilium. 4. Ischium. 5. Pubis. 6. Synsacrum. 7. Dorsal iliac fossa. 8. Acetabulum. 9. Antitrochanter. 10. Cranial border of preacetabular ilium. 11. Muscular lines. 12. Dorsal iliac crest 13. Lateral iliac crest. 14. Dorsolateral iliac crest. 15. Ventral border of postacetabular part. 16. Obturator foramen. 17. Ischiopubic fenestra. 18. Ischial crest. 19. Caudal part of ischium 20. ilio-ischiatic notch. 21. Pectineal process. 22. Pubic symphysis. 23. Iliosynsacral canal. 24. Medial surface of preacetabular ilium. 25.Caudal part of pubis

The Ostrich Pelvic girdle (Os coxae) was formed by three bones: the ilium, ischium and pubis (Fig. 4A). They contributed to the formation of the acetabulum (Fig. 4A). The mean weight of os coxae was 1180.00 ± 0.90 gm. The metric data of os coxae of ostrich were presented in Table 4.

**Table 4:** Metric data of os coxae (pelvic girdle) parts of ostrich (*Struthio camelus*)

Item of Description	Mean length in cm
**Preacetabular ilium**	10.00 ± 0.30
**Acetabular ilium**	6.00 ± 0.25
**Postacetabular ilium**	30.00 ± 0.27
**Ischium**	32.00 ± 0.20
**Pubis**	55.00 ± 030

**Fig. 5:**
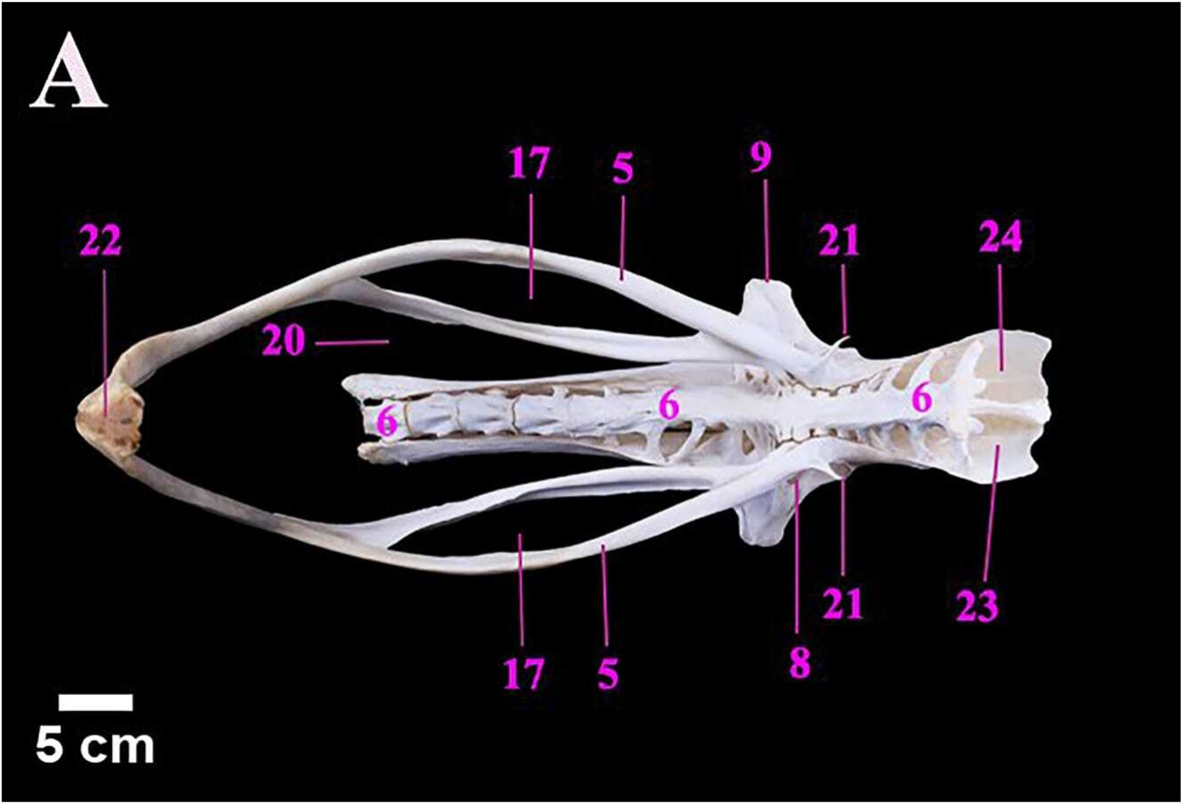
**A** showing ventral view of the pelvic girdle. Legend: 1. Preacetabular part of ilium. 2. Acetabular part of ilium. 3. Post acetabular part pf ilium. 4. Ischium. 5. Pubis. 6. Synsacrum. 7. Dorsal iliac fossa. 8. Acetabulum. 9. Antitrochanter. 10. Cranial border of preacetabular ilium. 11. Muscular lines. 12. Dorsal iliac crest 13. Lateral iliac crest. 14. Dorsolateral iliac crest. 15. Ventral border of postacetabular part. 16. Obturator foramen. 17. Ischiopubic fenestra. 18. Ischial crest. 19. Caudal part of ischium 20. ilio-ischiatic notch. 21. Pectineal process. 22. Pubic symphysis. 23. Iliosynsacral canal. 24. Medial surface of preacetabular ilium. 25.Caudal part of pubis

The ilia were flat bony plates forming the roof and sides of the hip bone. It was divided into three parts: preacetabular, acetabular and post-acetabular (Fig. 4A). The preacetabular part of ilium consisted of two surfaces: lateral and medial surfaces (Fig. 5A) and four borders: cranial, caudal, ventral and dorsal borders. The lateral surface was concave, rough and contained many muscular lines (Fig. 4A) and just cranial to acetabulum, it also possessed the dorsal iliac fossa (Fig. 4A). The dorsal border of each ilium fused dorsally, forming a prominent dorsal iliac crest (Fig. 4B) which diverged into two lines, dorsal to the acetabulum. Laterally, the free edge of the preacetabular part represented the lateral iliac crest (Fig. 4A). The cranial border was thin and concave in its middle (Fig. 4 A). The acetabular part of the ilium contained two surfaces: the medial and the lateral. The dorsal border of the acetabulum was projected forming the antitrochanter (Fig. 4A & B) which articulated with major trochanter of the femur. The post acetabular part was an elongated bony plate that contained two surfaces: the medial and the lateral surfaces and three borders: dorsal (thick), caudal (tuberous) and ventral (thin) borders. The dorsal borders of ilia of opposite sides were separated from each other by the spinous crest of synsacrum. The ventral border was concave, cranially and caudally, and convex in its middle. Dorsolaterally, the post acetabular part showed a dorsolateral crest of the ilium (Fig. 4A).

The ischium was formed of two narrow bony parts that diverged caudally. The ischium contributed to the formation of the acetabulum, cranially while its caudal part was thick and attached to the pubis. The obturator foramen was situated ventral to the cranial part of ischium and just caudoventral to acetabulum. This foramen also communicated with the pubio-ischiatic fenestra. On the lateral surface of the ischium, there was a prominent ischial crest along its length. The ischium was not attached caudally with the ilium therefore leaving a large ilio-ischiatic notch in between (Fig. 4A & B).

The Pubis was an elongated bone, that constituted the most caudoventral portion of the Os coxae. It contributed to the formation of the acetabulum, cranially and had a pectineal process ventral to the acetabulum (Fig. 4A & B). The pubic bones diverged caudally forming a “V-shaped” architecture with enlarged caudal ends (Fig. 4A) and joined to form a well-defined pubic symphysis (Fig. 5A).

**Fig. 6:**
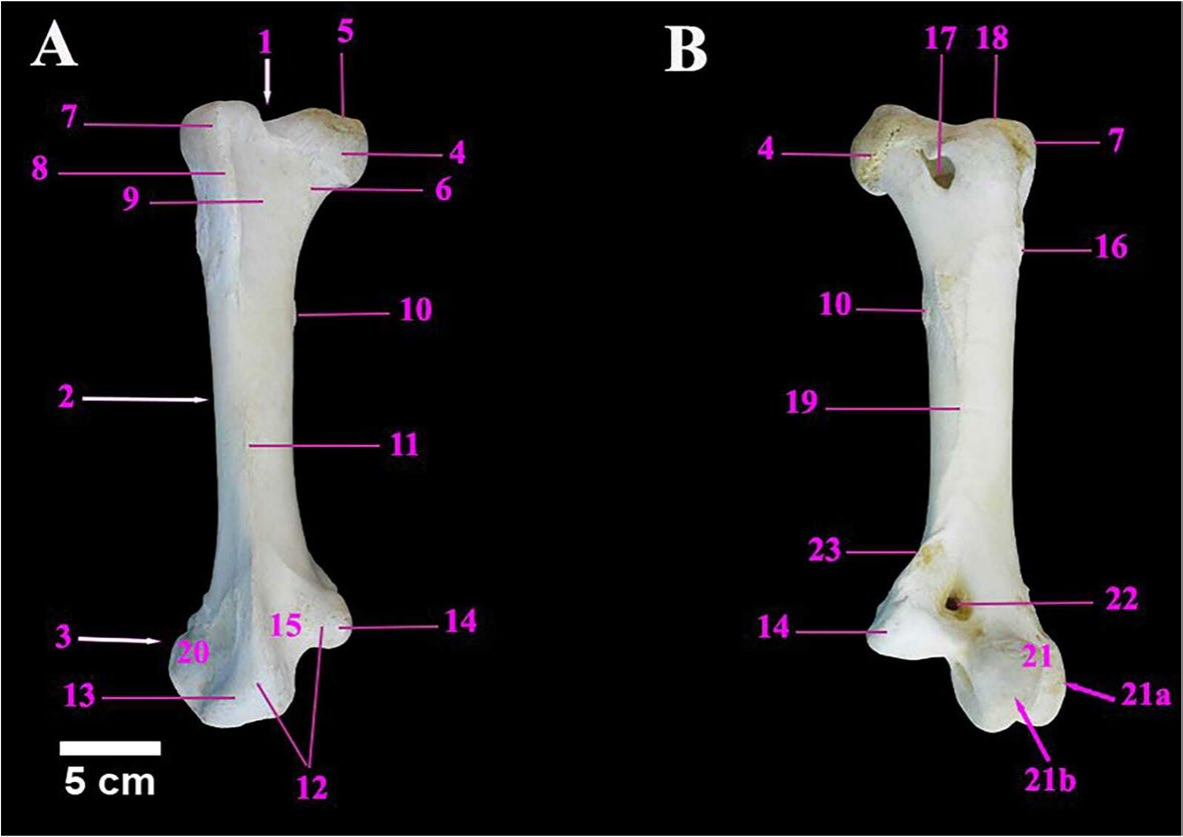
**A** & **B** Photographs showing the cranial and caudal view of the right femur of male Ostrich respectively. Legend: 1. Femur proximal extremity. 2. Femur Shaft. 3. Femur distal Extremity. 4. Head of femur. 5. Fovea capitis. 6. Neck of femur. 7. Trochanteric major. 8. Trochanteric ridge 9. Trochanteric fossa. 10. trochanteric minor. 11. Cranial intermuscular line. 12. Trochlea. 13. Lateral condyle. 14. Medial condyle. 15. Intercondylar sulcus (patellar sulcus). 16. rough area for muscle attachment. 17. Pneumatic foramen. 18. Antitrochanter articular surface. 19. Linea aspera (caudal intermuscular line). 20. Lateral femoral epicondyle. 21. Fibular trochlea. 21a. Lateral ridge of fibular trochlea. 21b. Medial ridge of fibular trochlea. 22. Supracondylar fossa. 23. Medial Supracondylar crest

The ostrich femur (thigh bone) was stout pneumatic and comparatively short bone. It was oriented obliquely forwards and downwards, and was present between the hip bone, proximally and the tibiotarsus bone, distally. It was shorter than the tibiotarsus bone. It had a shaft and two extremities. The proximal extremity was formed by the head, neck and great trochanter (Fig. 6A & B). The mean weight of the femur was 428.00 ± 1.15 gm. The metric data of the femur of ostrich was shown in Table 5.

**Table 5:** Metric data of femur bone of ostrich (*Struthio camelus*)

Item of Description	Mean length (cm)	Mean width (cm)	Mean circumference of the shaft (cm)
**Femur**	30.00 ± 0.23	11.00 ± 0.23 (P)	19.00 ± 0.17 (P)
		9.00 ± 0.25 (D)	14.50 ± 0.11 (MS)
		5.00 ± 0.20 (M)	17.00 ± 0.144 (D)

**Fig. 7:**
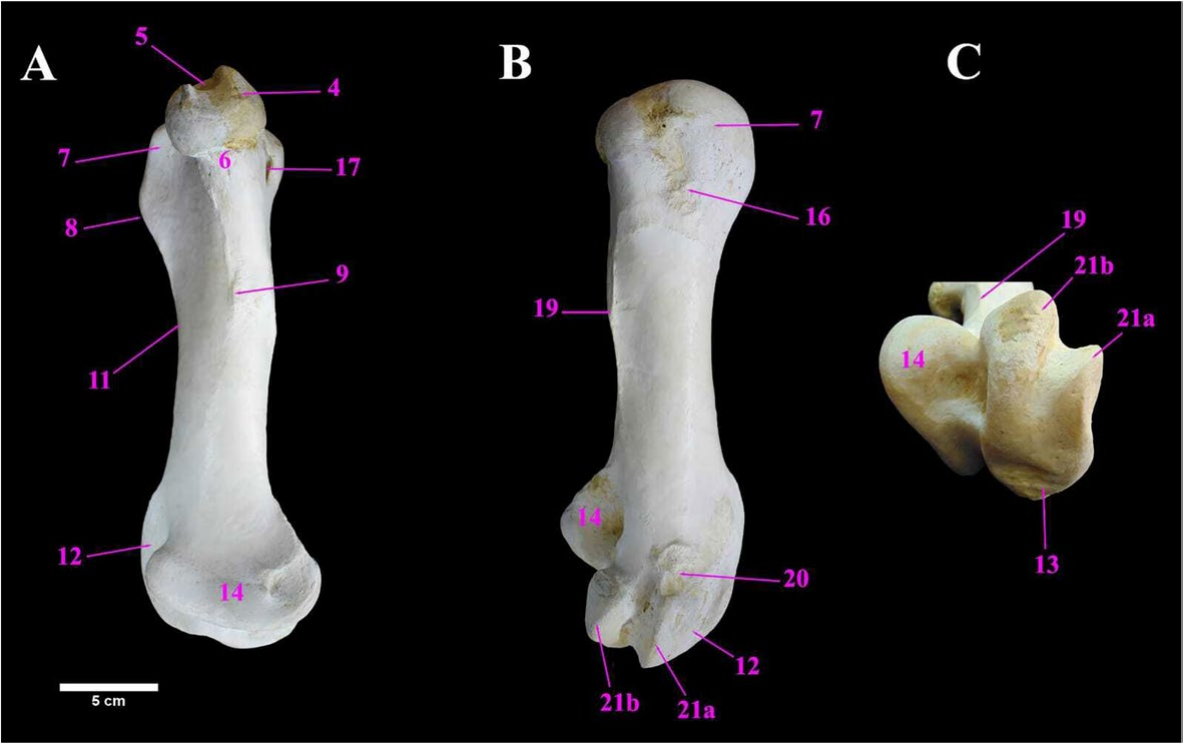
**A** & **B** Photographs showing the medial and lateral surfaces of the right femur of male Ostrich, respectively, **C** The ventral view of the femur distal extremity. Legend: 1. Femur proximal extremity. 2. Femur Shaft. 3. Femur distal Extremity. 4. Head of femur. 5. Fovea capitis. 6. Neck of femur. 7. Trochanteric major. 8. Trochanteric ridge 9. Trochanteric fossa. 10. trochanteric minor. 11. Cranial intermuscular line. 12. Trochlea. 13. Lateral condyle. 14. Medial condyle. 15. Intercondylar sulcus (patellar sulcus). 16. rough area for muscle attachment. 17. Pneumatic foramen. 18. Antitrochanter articular surface. 19. Linea aspera (caudal intermuscular line). 20. Lateral femoral epicondyle. 21. Fibular trochlea. 21a. Lateral ridge of fibular trochlea. 21b. Medial ridge of fibular trochlea. 22. Supracondylar fossa. 23. Medial Supracondylar crest

The femoral head was medially placed and projected above the level of the greater trochanter, with a small but deep Fovea ligamentum capitis. The neck was short and smooth. The Trochanter major was characterized by a sharp trochanteric ridge and a rough area for muscular attachments lateral to the trochanteric ridge, in addition to a shallow trochanteric fossa medial to the ridge, cranially. The trochanter minor was a rough area below the head. One of the most characteristic features of the femur in ostrich was the existence of a large pneumatic foramen ventrolateral to its head at caudal aspect (Fig. 6A & B). The femoral shaft had two borders; cranial and caudal and two surfaces; medial and lateral (Figs. 6A and 7A, B). The cranial intermuscular line and the linea aspera were detected as prominent ridges that were found along the cranial and caudal borders. The distal extremity of the femur had a large asymmetrical trochlea, cranially which was composed of a large lateral condyle and small medial condyle, separated by an inverted “V-shaped” intercondylar sulcus (patellar sulcus). The lateral condyle and lateral femoral epicondyles formed the fibular trochlea, caudally (Fig. 6A/21) which was divided into two ridges; the lateral ridge articulated with the head of fibula whereas the medial ridge articulated with the tibiotarsus. The supracondylar fossa was dorsomedial to the fibular trochlea and the supracondylar crest) was detected above the medial condyle (Figs. 6A, B and 7A, B, C).

**Fig. 8:**
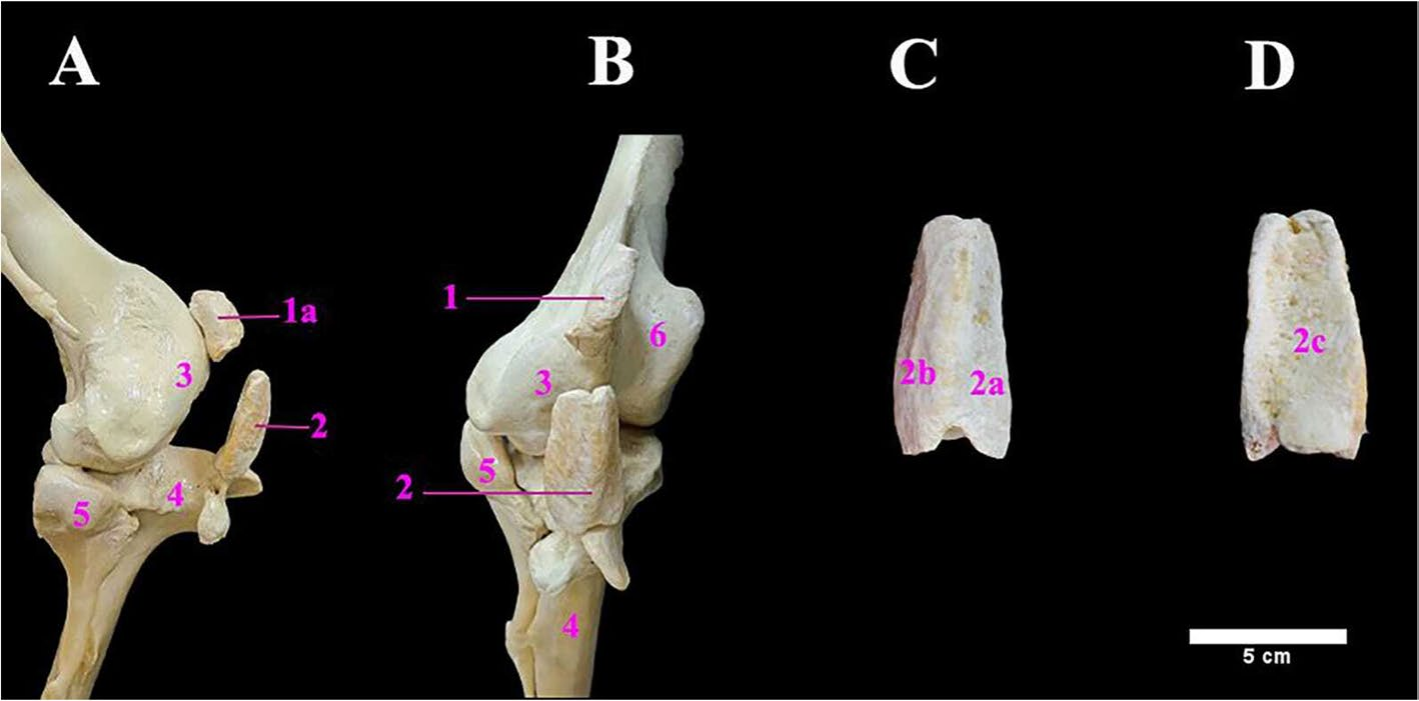
**A** & **B** Photograph showing the lateral and cranial views of the knee joint of male ostrich, respectively; **C** & **D** showing cranial and caudal views of the distal patella. 1. Proximal patella. 1a. Non articular surface of proximal patella. 2. Distal patella. 2a. Medial surface of distal patella. 2b. Lateral surface. 2c. Caudal surface. 3. Lateral condyle of Femur. 4. Tibial crest (patellar crest). 5. Fibular head. 6. Medial condyle of femur

Interestingly, the ostrich had double patellae; a proximal patella and a distal patella (Fig. 8A, B, C & D). The proximal patella appeared as a small wedge-shaped bone, sliding over the lateral condyle of the femur. It possessed 3 surfaces i.e., a single smooth articular surface and two rough non-articular ones. The distal patella was long, quadrilateral in outline, and was situated cranially to the lateral condyle of femur and above the tibial crest (patellar crest). It exhibited three surfaces viz., the lateral, medial, and rough caudal surfaces (Fig. 8C & D).

**Fig. 9:**
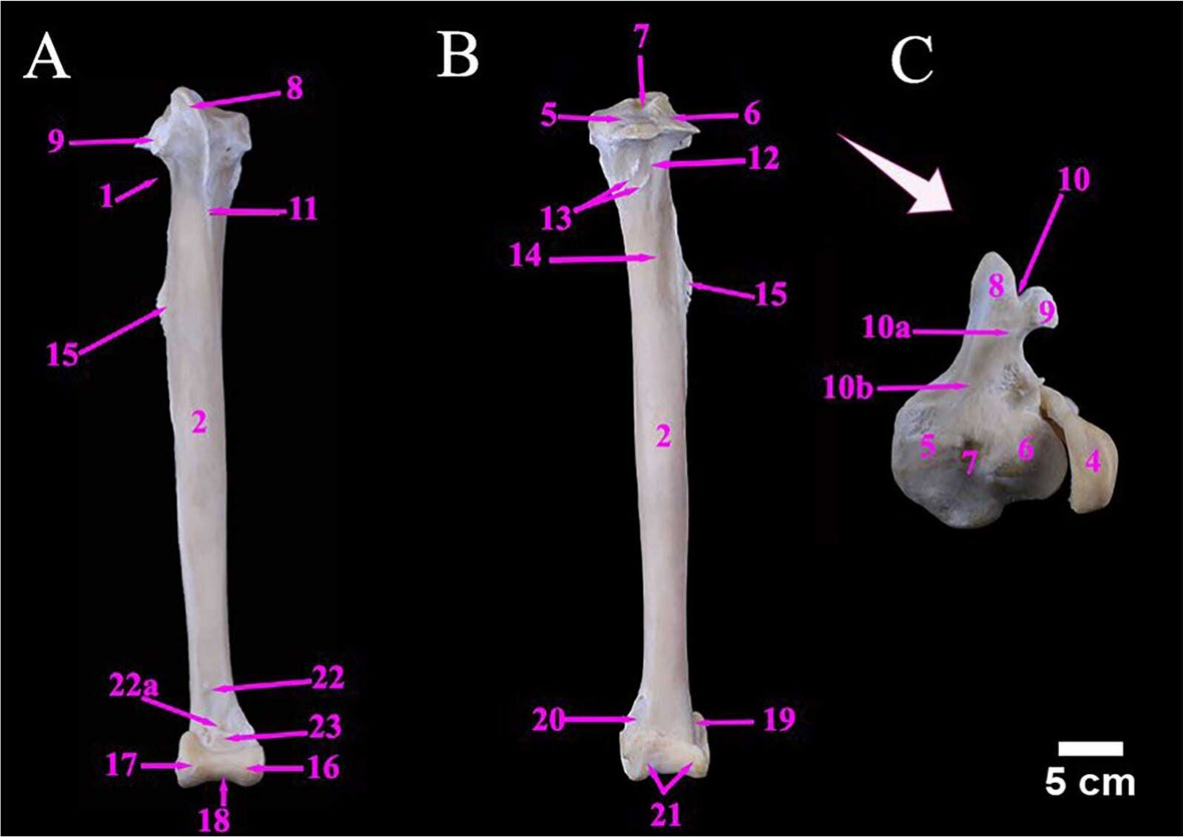
**A** & **B** Photograph showing cranial and caudal aspect of left tibiotarsus of male Ostrich, respectively. **C** the proximal end of left tibiotarsus. Legend: 1. Proximal extremity of tibiotarsus. 2. Tibiotarsus Shaft. 3. Distal extremity of tibiotarsus. 4. Fibula. 5. Medial condyle. 6. Lateral condyle. 7. Intercondylar fossa. 8. Cranial cnemial crest. 9. Lateral cnemial crest. 10. Intercnemial sulcus. 10a. Patellar crest (Tibial crest). 10b. Retropatellar fossa 11. Extensor line. 12. Flexor fossa. 13. Muscular lines. 14. Popliteal line. 15. Fibular crest. 16. Medial distal condyle. 17. Lateral distal condyle. 18. Sulcus cartilaginous tibialis. 19. Lateral epicondyle. 20. Medial epicondyle. 21. Trochlea cartilaginis tibialis. 22. Extensor Sulcus. 22a. Extensor canal. 23. Supratendinal Bridge. 24. Caput fibulae. 25. Fibular articular facet. 26. Spina fibulae. 27. Tubercle. 28. Proximal interosseous foramen. 29. Distal interosseous foramen

**Fig. 10:**
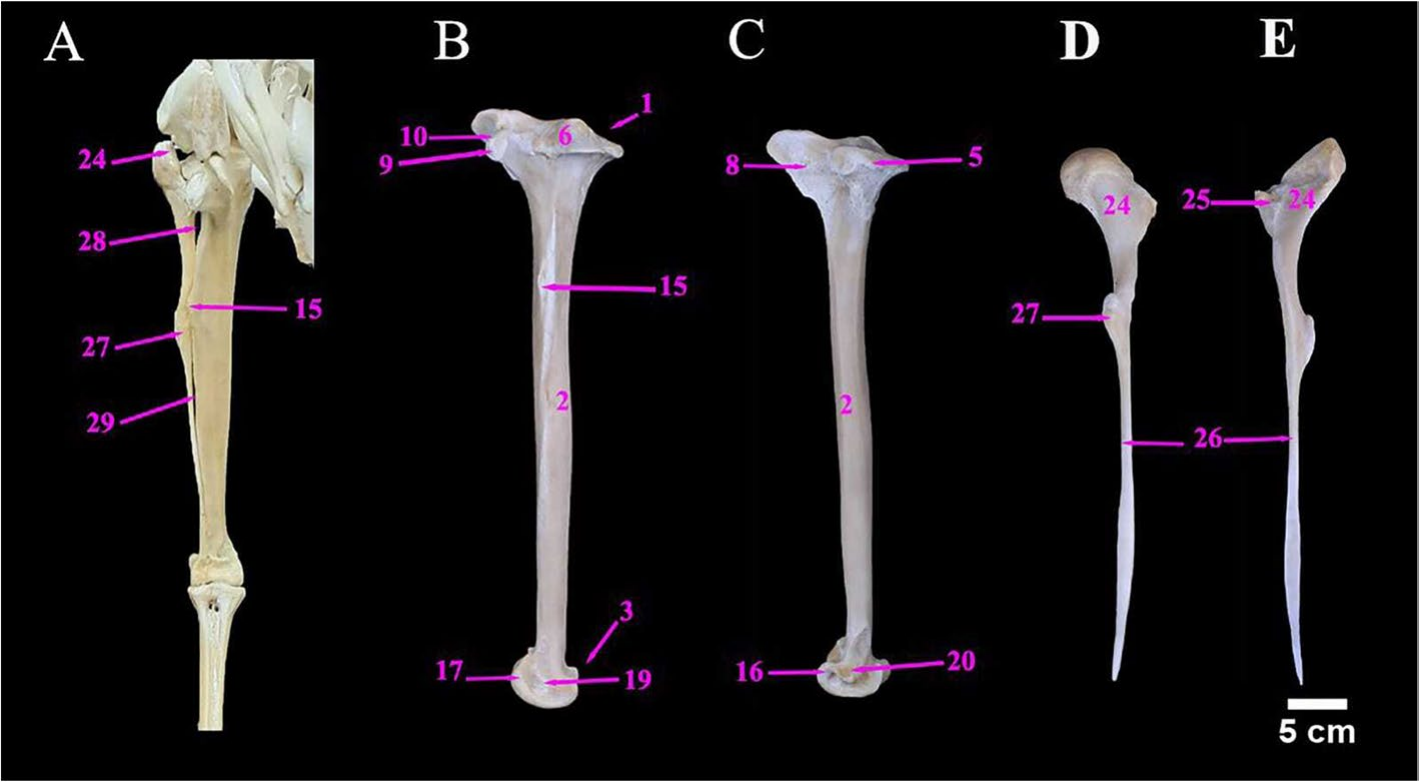
**A** The cranial view of the right tibiotarsus and fibula of male Ostrich, **B** & **C** Photographs showing lateral and medial aspects of left tibiotarsus respectively. **D** & **E** lateral and medial aspect of the left fibula. Legend: 1. Proximal extremity of tibiotarsus. 2. Tibiotarsus Shaft. 3. Distal extremity of tibiotarsus. 4. Fibula. 5. Medial condyle. 6. Lateral condyle. 7. Intercondylar fossa. 8. Cranial cnemial crest. 9. Lateral cnemial crest. 10. Intercnemial sulcus. 10a. Patellar crest (Tibial crest). 10b. Retropatellar fossa 11. Extensor line. 12. Flexor fossa. 13. Muscular lines. 14. Popliteal line. 15. Fibular crest. 16. Medial distal condyle. 17. Lateral distal condyle. 18. Sulcus cartilaginous tibialis. 19. Lateral epicondyle. 20. Medial epicondyle. 21. Trochlea cartilaginis tibialis. 22. Extensor Sulcus. 22a. Extensor canal. 23. Supratendinal Bridge. 24. Caput fibulae. 25. Fibular articular facet. 26. Spina fibulae. 27. Tubercle. 28. Proximal interosseous foramen. 29. Distal interosseous foramen

The ostrich Tibiotarsus was formed by the fusion of the tibia and proximal row of the tarsal bones. Mean weight of tibiotarsus was 960.00 ± 1.08 gm. The metric data of Tibiotarsus of ostrich was shown in Table 6. It was directed caudoventrally in an oblique manner. The proximal extremity contained two prominent condyles namely the medial, and the lateral condyles which were separated by intercondylar fossa (Figs. 9B, C & 10C). The fibula was a long bone that lay along the lateral surface of the tibiotarsus and ended before its distal extremity. The metric data of fibula of ostrich was presented in Table 6.

**Table 6:** Metric data of tibiotarsus and fibula bones of ostrich (*Struthio camelus*)

Item of Description	Mean length (cm)	Mean width (cm)	Mean circumference of the shaft (cm)
**Tibiotarsus**	52.00 ± 0.50	4.50 ± 0.31 (M)	14.00 ± 0.20 (P)
		5.20 ± 0.25 (P)	11.50 ± 0.15 (MS)
		3.50 ± 0.15 (D)	6.00 ± 0.20 (D)
**Fibula**	44.00 ± 0.41	1.32 ± 0.28(M)	1.72 ± 0.24(M)

The fibula consisted of the head (caput fibulae) and a pointed long shaft. The fibular articular facet was located cranially below the caput fibulae for articulation with the lateral condyle of the tibiotarsus. The proximal and distal interosseous foramina were located between the fibula and tibiotarsus, proximally and distally, respectively (Fig. 10A, D & E).

The tibiotarsus cranially possessed the larger cranial cnemial crest and the lateral cnemial crest and the latter crest extended distally as an extensor line (Fig. 9A), and they were separated by the intercnemial sulcus. The patellar crest was an oblique crest connecting the two latter crests, proximally (Fig. 9A & C). The Shaft had three surfaces in the proximal half and two surfaces in the distal half. Caudally, the shaft had a shallow flexor fossa and two muscular lines, just below the lateral condyle (Fig. 9B). The Fibular crest was a linear crest on the lateral aspect of the proximal third of tibiotarsus shaft for articulation with fibula (Fig. 10A). The distal extremity of the tibiotarsus had a well-developed trochlea, caudally and two condyles (lateral and medial condyles), cranially for articulation with the tarsometatarsus. Two small epicondyles i.e., lateral and medial epicondyles were also noticed. The distal end of the tibiotarsus shaft had a deep extensor sulcus, cranially which led to the extensor canal and was formed by the bony supratendinal bridge (Figs. 9A, B, C, 10B & C).

The tarsometatarsus was a strong long bone formed by the fusion of the 2^nd^, 3^rd^ and 4^th^ metatarsal bone (II, III, IV) and the distal row of tarsal bones however, the 2^nd^ metatarsal was externally lost in adults. Mean weight of the tarsometatarsus is 510.00 ± 0.57 gm. It extended obliquely in cranioventral direction. The metric data of ostrich tarsometatarsus was shown in Table 7.

**Table 7:** Metric data of tarsometatarsus bone of ostrich (*Struthio camelus*)

Item of Description	Mean length (cm)	Mean width (cm)	Mean circumference of the shaft (cm)
**Tarsometatarsus**	46.00 ± 0.28	3.50 ± 0.15 (M)	14.00 ± 0.25 (P)
		4.00 ± 0.23 (P)	10.00 ± 0.30 (MS)
		3.00 ± 0.15 (D)	11.00 ± 0.32 (D)

**Fig. 11:**
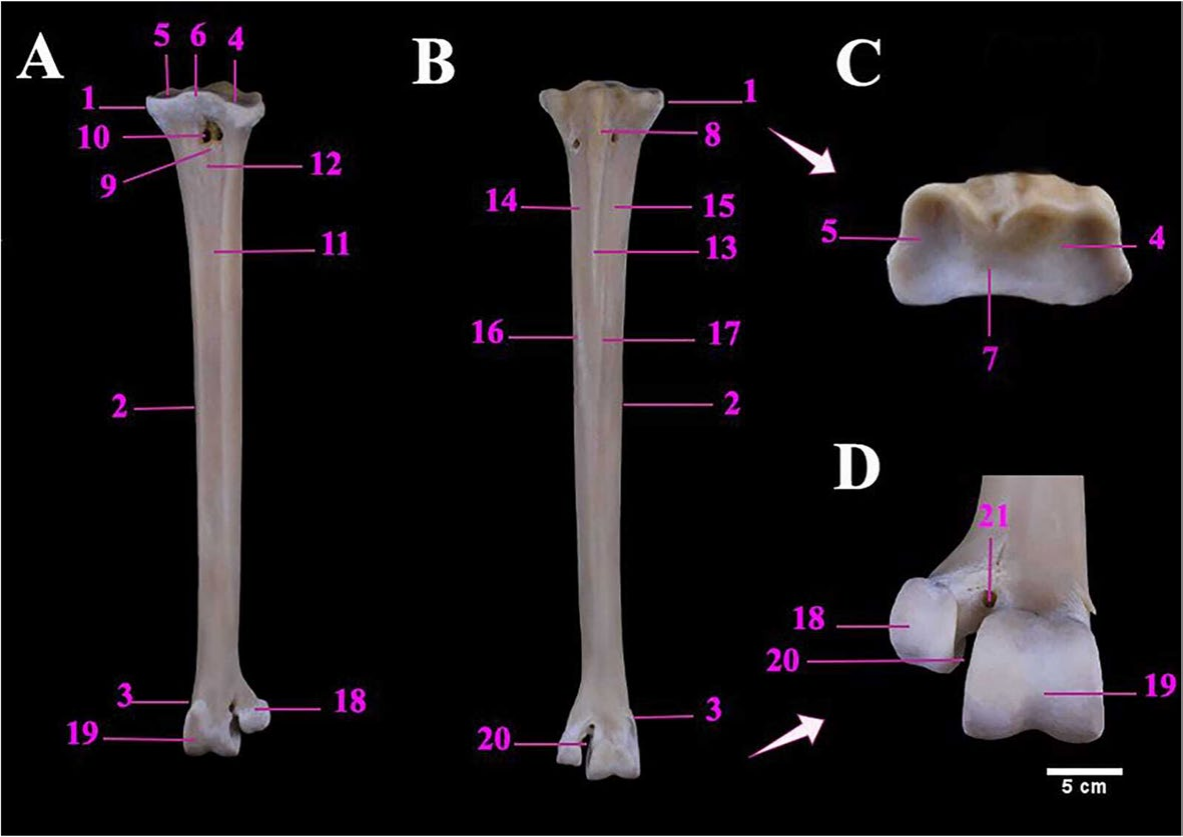
**A** & **B** Photographs showing the dorsal and planter surface of the left tarsometatarsus bone of male Ostrich respectively, **C** proximal articular surface and **D** the distal articular surface of the left tarsometatarsus. 1. Proximal extremity. 2. Shaft. 3. Distal extremity. 4. Lateral cotyla. 5. Medial cotyla. 6. Intercotylar eminence. 7. Ligamentous sulcus. 8. Hypotarsus. 9. Dorsal infracotylar fossa. 10. Proximal vascular foramina. 11. Longitudinal extensor sulcus. 12. Crista tibialis cranialis. 13. Crista medianoplantaris. 14. Lateral flexor sulcus. 15. Medial flexor sulcus. 16. Lateral planter crest (Crista plantares lateralis). 17. Medial planter crest (Crista plantares medialis). 18. Fourth metatarsal articular trochlea (Lateral trochlea). 19. Third metatarsal articular trochlea (Medial trochlea). 20. Intertrochlear notch. 21. Distal interosseous canal

The proximal extremity of the tarsometatarsus showed lateral and medial cotyla which were separated dorsally by the intercotylar eminence and proximally by a transverse groove caudal to the eminence called the ligamentous sulcus. On the dorsal surface of the proximal extremity, there was a deep depression called the infracotylar fossa containing two proximal vascular foramina and crista tibialis cranialis. The hypotarsus was a bony ridge present proximally on the planter surface of the tarsometatarsus which continued distally as a bony crest i.e., the crista medianoplantaris. The hypotarsus was surrounded from both sides by a shallow groove for flexor tendons of the toe viz., lateral and medial flexor sulcus. The hypotarsus was flanked distally by two bony crests: lateral and medial plantar crests (Fig. 11A, B & C).

The shaft of the tarsometatarsus had a longitudinal groove for extensor tendons named longitudinal extensor sulcus. The distal extremity of the tarsometatarsus was characterized by the presence of two prominent trochleae: lateral and medial trochlea separated by a deep intertrochlear notch that carried the distal interosseous canal. The lateral trochlea (fourth metatarsal articular trochlea) was small and articulated with the fourth digit (IV) but the medial trochlea (third metatarsal articular trochlea) was large and extended more distally and articulated with the third digit (III) (Fig. 11A, B, C & D).

**Fig. 12:**
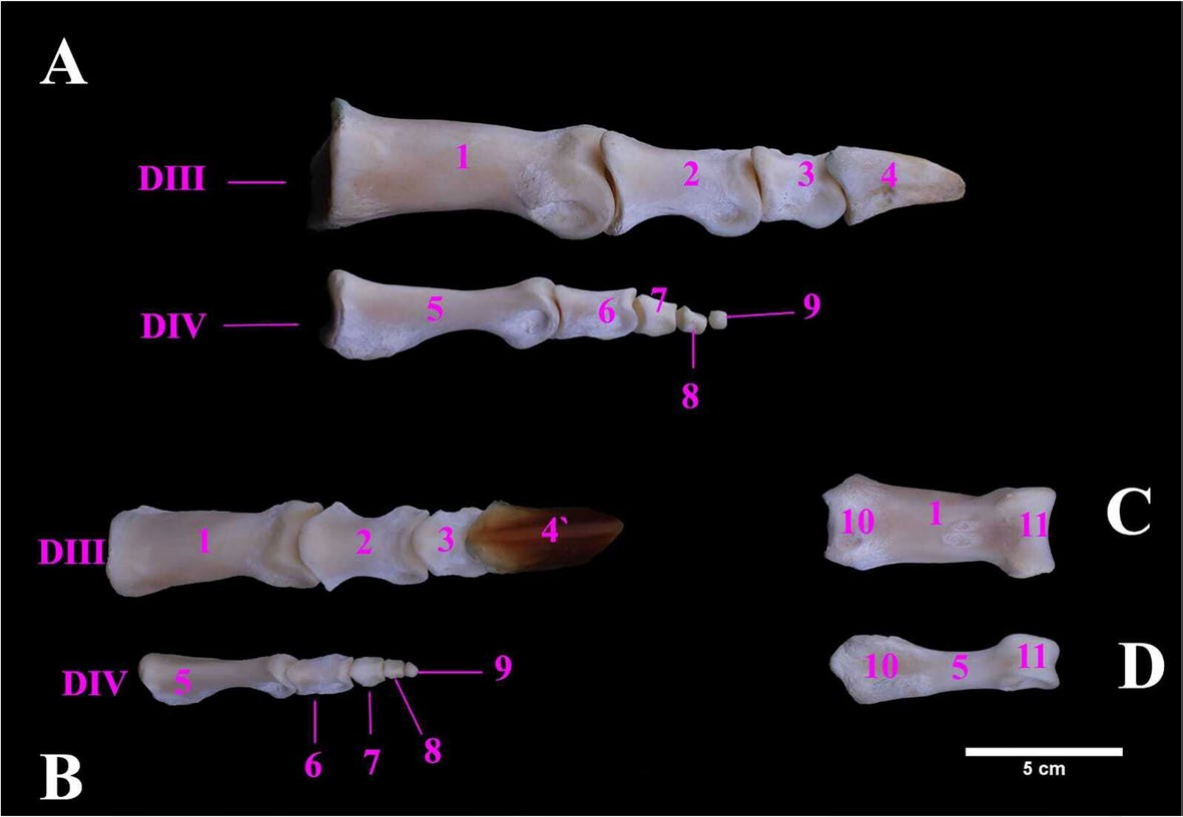
**A** & **B** Photographs showing the lateral and dorsal surfaces of male Ostrich right pedal digits, **C** & **D** the planter surfaces of 1st phalanges of digits III and IV. DIII. Third pedal digit. DIV. Fourth pedal digit. 1. First phalanx of III digit. 2. Second phalanx of III digit. 3. Third phalanx of the III digit. 4. Fourth phalanx of III digit. 4`. Fourth phalanx of the III digit is covered with its claw. 5. First phalanx of IV digit. 6. Second phalanx of IV digit. 7. Third phalanx of IV digit. 8. Fourth phalanx of IV digit. 9. Fifth phalanx of IV digit. 10. Proximal extremity. 11. Distal extremity

The ostrich pedal digits were only two; the third (III) and fourth (IV) digits (Fig. 12A). The third digit was larger and longer than the fourth one. The metric data of pedal digits of ostrich were tabulated (Table 8). The third digit was composed of four phalanges: the 1^st^, 2^nd^, 3^rd^ & 4^th^ phalanges. The 4^th^ phalanx was the smallest and concealed within a horny structure called a claw. The proximal extremity of the first phalanx was articulated with the tarsometatarsus while its distal extremity articulated with the second phalanx of digit III. The fourth digit (had five phalanges namely 1^st^, 2^nd^, 3^rd^, 4^th^ and 5^th^. The fifth one was very small. The 1^st^ phalanx of digit IV) was smaller in size than the 1^st^ phalanx of digit III (Fig. 12A, B, C & D).

**Table 8:** Metric data of pedal digits of ostrich (*Struthio camelus*)

Item of Description		Mean width (cm)	Mean length (cm)	Mean width (cm)
**Digit III**	1^st^ phalanx	70.00 ± 0.40	9.00 ± 0.14	5.00 ± 0.34
	2^nd^ phalanx	26.00 ± 0.38	5.00 ± 0.20	3.00 ± 0.38
	3rd phalanx	10.00 ± 0.36	3.00 ± 0.21	3.00 ± 0.30
	4^th^ phalanx with claw	22.00 ± 0.29	7.00 ± 0.18	3.50 ± 0.14
	4th phalanx with claw	15.00 ± 0.57	5.00 ± 0.29	2.50 ± 0.20
	1^st^ phalanx	20.00 ± 0.78	7.50 ± 0.15	2.00 ± 0.18
**Digit IV**	2^nd^ phalanx	4.00 ± 0.88	3.00 ± 0.23	1.50 ± 0.32
	3^rd^ phalanx	2.00 ± 0.50	1.50 ± 0.29	1.20 ± 0.20
	4^th^ phalanx	0.50 ± 0.42	1.00 ± 0.22	0.80 ± 0.37
	5^th^ phalanx	0.20 ± 0.45	0.50 ± 0.16	0.60 ± 0.13

## Discussion

In the present study, the clavicle was not observed in ostrich which was found in accordance of earlier reports [6], and [7] but in contrast to other reports in ostrich where it was noticed [8]. The clavicular absence resulted in the formation of supracoracoideus sulcus in the place of triosseal canal reported earlier [9–11].

The large pneumatic foramen was present on the medial surface of scapula in ostrich whereas it was present at the medial surface of the acrocoracoid process in domestic pigeons [12]. Moreover, multiple pneumatic foramina were seen in the coracoid bone of ostrich [8]. The coracoid bone had sternal and scapular wings as also observed in in avialans [13], however, an additional acrocoracoid process was also observed in present study. Whereas the lateral and medial angular processes of coracoid bone were mentioned in emu [14].

The present observation on the length of wing in the ostrich was concurred with earlier description in ostrich [7]. The pneumotricipital fossa in ostrich showed several pneumatic foramina and was similar to the observations in chickens [4], but was also in contrast with the earlier reports in ostriches [7] and [8]. The distal extremity of humerus showed medial and lateral condyles of nearly equal size. This observation mimicked the reports in emu [14] but challenged the earlier observations in ostriches [7] where only a single condyle was noticed.

The Radius and ulna were separated by the interosseous space and was in resemblance with similar findings in emu [14]. The ulna was longer than radius in present study and was in agreement with the observations in Southern Cassowary [15]. However, these bones were of an equal length in emu [14]. The distal extremity of the ulna had a single carpal trochlea as also described earlier [8].

The mean length of scapula in present study was 15.00 ± 0.23 cm, which was 16.40 ± 0.24 cm in emu [16]. The mean length of Coracoid (sternal wing) was 10.00 ± 0.17 cm, which was 6.70 ± 0.12 cm in emu. The humerus was 33.00 ± 0.46 cm long in ostrich observed in present investigation but it was 40.00–43.00 cm and 10.00 cm in earlier observations on humerus of ostrich and in emu, respectively [14]. In our study, the mean width of proximal extremity of humerus was 2.60 ± 0.35 cm whereas it was reported 1.90 cm in emu [14]. The carpometacarpus bone in present study was formed by the fusion of the distal row of carpal bones and three 1^st^, 2^nd^ and 3^rd^ metacarpal bones similar to earlier findings in emu [17] and ostrich [7]. However, in the chicken the 2^nd^, 3th and 4^th^ metacarpal bones were involved in similar formation [4] and [8].

The digits of the wing included the first, second, and third digits as also described in emu [14] and in Southern Cassowary [15] while in chicken, the digits found in wing were the second, third, and fourth digits [4]. The major digit was the largest and consisted of two phalanges and the minor digit contained only one phalanx as also reported earlier [15] and [7], respectively. The ilium was composed of preacetabular, acetabular, and post acetabular parts similar to the description in emu [18], however, the previous observation on the ilium of ostriches quoted to have two parts only [19] and [20]. The acetabulum was circular, centrally perforated bony ring was concurred with earlier reports [18].

The preacetabular ilium in present study was consisted of two surfaces and four borders as also described earlier [18]. The dorsal border of each ilium fused dorsally to form a prominent dorsal iliac crest as also described in chicken [4] and ostrich [19]. The lateral iliac crest was represented by the lateral free edge of the preacetabular part. The post acetabular part was an elongated bony plate in present study which was in contrast to the previous observations in ostrich and emu where it was described as triangular in shape [1].

The ischium in ostriches had two narrow bony parts and was in contrast to the triangular outline in domestic fowl and duck [1]. Similar to previous observations in ostrich [7], the obturator foramen was placed just caudoventral to acetabulum in present investigation. The pubic bones had pubic symphysis caudally and was supported by earlier reports [19] but was contrary to the observation in emu [1]. The length of ilium was 46.00 cm in present study, however, previous reports claimed to have 60.00 cm [7], 50.50 ± 0.70 cm in ostrich, and 40.20 ± 0.40 cm in emu [1]. The length of pubis was 55.00 ± 0.29 cm in ostrich in our study while it was mentioned to be 64.00 cm in ostrich [7].

The current study also revealed that the femoral head was projected above the level of the greater trochanter and was contrary to the observations in emu [3] and chicken [4]. A large pneumatic foramen was also seen in femur in our study which was similar to the previous reports in emu [3].

The distal extremity of femur had trochlea, cranially and was in accordance to findings in hill fowl of Uttarakhand [21]. The fibular trochlea was formed by the lateral condyle and lateral femoral epicondyles were divided into two ridges; the lateral ridge and the medial ridge as also described in chicken [4] and ostrich [22]. The femoral length was 30.00 ± 0.23 cm and was in accordance to previous claims in ostrich [7] and [1] while it was 20.00 cm in emu [3]. The length of tibiotarsus was 52.00 ± 0.50 cm in our study however it was 53.80 ± 0.40 cm [7] and 55.00 cm in ostrich, 43.70 ± 0.30 cm in emu [1]. The length of the tarsometatarsal was 46.00 ± 0.28 cm in ostrich while it was reported to be 44.80 ± 0.40 cm in ostrich and 40.20 ± 0.30 emu [1].

Two patellae (proximal and distal) were noticed in present investigation. The proximal one was similar to the single patella found in other species, while the distal one was in the form of a prominent bony process which was firmly adhered to the proximal extremity of the tibiotarsus.

The couple of patellae were also mentioned earlier in ostrich [22] and [23]. On the other hand, only a single patella was mentioned in ostrich [1] and chicken [4] while the patella was absent in ratites [2]. The existence of two patellae in ostrich may be helpful in protection of the tendons against laceration and damage in these heavy but fast-running birds.

Tibiotarsus was formed by the fusion of the tibia and proximal row of tarsal bone as also reported in Indian eagle owl [24]. It showed cranial and lateral cnemial crest which tallied the observations in ostriches [1, 7, 22]. The distal end of the tibiotarsus had a deep extensor sulcus leading to the extensor canal as also described in hill fowl of Uttarakhand [21]. It also possessed a well-developed trochlea, caudally as also described earlier [1] and [24]. The fibula was long bone placed along the lateral surface of the tibiotarsus, and ended just before its distal extremity similar to previous reports [24].

The tarsometatarsus was formed by the fusion of the metatarsal bones (II, III, IV) and the distal row of tarsal bones as also reported earlier [25]. However, a single report on the presence of the fist metatarsal in ostrich was found [1] but it was not noticed in emu [26]. The hypotarsus was a single bony ridge, proximally on the planter surface of the tarsometatarsus as also mentioned in earlier reports [1]) and [26]. distal extremity of the tarsometatarsus had two prominent trochleae: lateral and medial trochlea as also claimed previously [1] and [20] but conversely three trochleae were described ostrich [7] and emu [26]The pedal digits were the third (III) and fourth (IV) digits in present study and was agreed with observations in other studies on ostrich [27] and [28]. The third digit was larger and longer than the fourth one, and it was constructed by four phalanges as also described earlier [27].

## Conclusion

The appendicular skeleton in Ostrich consisted of shoulder girdle, wings, pelvic girdle, and pelvic limbs. The shoulder girdle was composed of the scapulo-coracoid while the clavicles could not be noticed. The triosseal canal was absent. The wing comprised the humerus, radius, ulna, radiale, ulnare, carpometacarpus, and digits. The Carpometacarpus was formed by the fusion of the distal row of carpal bones and three metacarpal bones. Digits of the wing were the alular, major, and minor digits.

The os coxae included the ilium, ischium, and pubis. The ischium was formed of two narrow bony pars that diverged caudally. Pubis was an elongated cylindrical bone, that constituted the most caudoventral portion of the os coxae. The femur was a stout short bone and was shorter than the tibiotarsus bone. The patellae were two in number viz., the proximal patella and distal patella. Tibiotarsus was formed by the fusion of the tibia and proximal row of the tarsal bone, it also articulated with fibula, laterally. The tarsometatarsus was a strong long bone formed by the fusion of the metatarsal bones (II, III, IV) and the distal row of tarsal bones. The ostrich pedal digits were only two; the third (III) and fourth (IV) digits. The third digit was larger and longer than the fourth one. The third digit was constructed by four phalanges while the fourth digit had five phalanges.

Our morphometric study in ostrich skeletons may help veterinarians to have a guideline for bone fracture diagnosis in ostrich. Our data may also help in the comparative anatomy of ostrich skeletons with other bird species.

## Data Availability

The skeleton of ostrich was mounted in the museum of the department of anatomy and embryology, faculty of veterinary medicine, Cairo university. The datasets are available from the corresponding author on reasonable request.

## Abbreviations

P: Proximal
D: Distal
M: Middle
m: Meter
MS: Midshaft
cm: Centimeter
gm: gram
